# Transitioning to quick response codes for patient information leaflet delivery.

**DOI:** 10.1016/j.rcsop.2025.100563

**Published:** 2025-01-10

**Authors:** Githa Singh, Sarel J. Brand, Vanessa Steenkamp

**Affiliations:** University of Pretoria Department of Pharmacology, Faculty of Health Sciences, Pretoria, South Africa

**Keywords:** Electronic patient information leaflet, Pharmacists, Quick response codes, Patient information leaflet, Patients, Pharmacy

## Abstract

**Background:**

The inclusion of a patient information leaflet (PIL) in medicine packaging is a legal requirement in most countries.

**Objective:**

To evaluate the feasibility of using quick response (QR) codes for electronic patient information leaflet (ePIL) delivery.

**Method:**

A mixed-method study based on surveys was conducted at the Tshwane District Hospital in South Africa. The demographics, ability and willingness of patients (330) and pharmacy staff (16) to scan a QR code for a commonly prescribed medicine was captured. A focus group study among 18 regulatory affairs pharmacists gauged their perception of ease of implementation of QR codes.

**Results:**

Of the 330 patients, most were 26–55 years of age (67 %) and 70 % were female. Irrespective of patient age and gender, >80 % were willing/ able to scan the QR code and preferred the ePIL (35 %) or ePIL with a hardcopy (45 %). Patients (>96 %) found it easy to read the ePIL (*C* = 0.487, *p* < 0.001) and locate the information sought (*C* = 0.521, *p* < 0.001). This sentiment was shared by dispensing pharmacy staff: easier to read (*C* = 0.746, *p* < 0.05) and locate information (*C* = 0.630, *p* < 0.05), with 69 % preferring either the ePIL or ePIL with a hardcopy. All the regulatory affairs pharmacists preferred the ePIL and indicated that it was easy to create a QR code for ePILs.

**Conclusion:**

Patients, dispensing pharmacy staff, and regulatory affairs pharmacists are willing to transition to ePILs. This makes going green and updating information in real time possible.

## Highlights

• ePILs have the potential to revolutionise the healthcare industry.
• ePILs will impact on how patients and healthcare professionals access medicine information.
• ePILs allow for the updating of new information in real time.

## 1 Introduction

The inclusion of a patient information leaflet (PIL) in the medicine packaging is a legal requirement in the majority of countries in the world, including South Africa.^1^ The PIL is critical to ensure the safe and effective use of the prescribed medicine by providing the patient with the latest product information.^2^ The advancement in technology and digitalisation has resulted in some countries implementing an electronic patient information leaflet (ePIL) using a quick response (QR) code.3., 4., 5., 6 Electronic patient information leaflets have been instituted in countries such as Japan, India and Australia (for non-prescription products). Some countries in the European Union, Canada, Brazil and the United States have implemented a dual system providing both the PIL and ePIL.3., 4., 5., 6, 7. There are many advantages to using ePILs, and these include quick, easy access to product information; the ability to rapidly update product safety information; improved readability due to the ability to increase font size and brightness; improved production timelines and a reduction in the use of paper.^8^ However, the current regulations in South Africa do not allow for the use of ePILs.^1^

Quick response codes were developed in 1994.^9^ The main advantage of a QR code is the ability to encode large amounts of information in a small two-dimensional space, which can be downloaded rapidly using a QR code scanner or mobile scanning device.^9^^,^^10^ The use of QR codes has expanded to areas such as commercial tracking, entertainment, in-store product labelling, and applications that are aimed at smartphones and other mobile devices.^9^, ^10^, 11. QR codes can be self-created using QR generator software available online; some at no cost, making it possible for companies to embrace QR coding to create an ePIL.^10^

## 2 Objectives

This study aimed to assess the feasibility of using QR codes for patient information delivery at a general outpatient department in the Tshwane district of Gauteng, South Africa. To achieve this, the ability and willingness of patients and dispensing pharmacy staff to scan a QR code to access an ePIL and their preference for an ePIL versus a printed PIL was assessed. A focus group study among regulatory affairs pharmacists, aimed to gauge their perception on the ease of implementation of QR codes linked to the ePIL.

## 3 Method

### 3.1 Study design

A mixed method design was employed, involving two cross-sectional quantitative surveys and a qualitative focus group study. The surveys were carried out among patients and dispensing pharmacy staff at Tshwane District Hospital Outpatient Department between 1 November 2022 and 30 June 2023. The focus group study was carried out among regulatory affairs pharmacists during the same period. The focus group was facilitated by the researcher, a qualified pharmacist. Face and content validity of the surveys and focus group questions was reviewed by subject matter experts. These included regulatory affairs pharmacists and family physicians. Ethics approval was received from the Faculty of Health Sciences, Research Ethics Committee (444/2022), University of Pretoria on 28 September 2022.

### 3.2 Data collection

Patients over the age of 18 and who could converse in English were included in the study. The Outpatient department has approximately 1810 patient consultations per month. To ensure a 95 % confidence interval with a 5 % precision, the sample size was estimated at 333 patients. A total of 333 patients volunteered to take part in the survey. The demographic information captured included age, gender and schooling level. Access to technology (device and data) was noted. The survey made use of a questionnaire which was answered after scanning the QR code of a commonly prescribed medication, ibuprofen. Ease of reading, location of information on the ePIL and preference for the ePIL versus hardcopy PIL were assessed.

The pharmacy survey was conducted among 17 dispensing pharmacy staff who volunteered to take part in the study. For this group; demographics, ability and willingness to scan the QR code and utilization of the ePIL to counsel patients was assessed (similar to that of the patient group). Ease of reading, location of information on the ePIL and preference for the ePIL versus hardcopy PIL was also assessed. To prevent response bias no open-ended questions were included in the survey.

The focus group study gauged regulatory affairs pharmacists' (18) familiarity of QR code and their perception on the ease of implementing QR code for ePILs. To eliminate bias, volunteer sampling was used. The participants contacted the investigator directly and a 2-h online meeting utilizing Microsoft Teams was set up with the focus group. An interview guideline with set questions was utilized to guide the researcher and enable capturing of notes for content analysis.

### 3.3 Data analysis

The data collected was analyzed using the Statistical Software Package developed for the Social Sciences (IBM® SPSS®, Version 29). Demographic data were summarized using descriptive statistics such as frequency and percentage. Cross-tabulation served to summarize the responses to observed categorical (nominal/ordinal) data in frequency and percentages. Contingency tables were completed to display the data where each row represents a category for one variable and each column represents a category for another variable.

Pearson's Chi-square test (*χ*^2^) test of independence was used to determine whether there was a significant relationship between two nominal (categorical) variables in the patient survey, whilst Fisher's exact test was used to determine whether there was a significant relationship between two nominal (categorical) variables in the pharmacy survey.

In all cases, a *p*-value <0.05 was considered significant. The measure of association was conveyed using the contingency coefficient, *C*, to indicate the strength of the relationship. When interpreting contingency coefficients, “0” was considered as zero association, “<0.10” as a weak association, “0.11–0.30” as a moderate association, “>0.31” as a strong association and “1” as perfect association.^12^

Reliability was assessed using Cronbach's alpha for the different sections of the survey, utilizing questions that were categorical, list or Likert scale. A Cronbach's alpha value of >0.70 indicates the data is reliable and demonstrates internal consistency.^13^ In the patient survey, questions related to; ‘reading the PIL’, ‘access to technology’ and ‘ease of reading and locating the information on the ePIL’ was utilized to calculate Cronbach's alpha. In the pharmacy survey questions related to; ‘access to technology’ and ‘ease of reading and locating the information on the ePIL’ was utilized to calculate Cronbach's alpha.

## 4 Results

### 4.1 Patient and pharmacy group

#### 4.1.1 Demographics of the population groups

The number of participants in the patient survey was 333, however 3 surveys had to be disregarded due to participants providing duplicate responses to questions. The demographics of the patients related to age, gender and schooling level are provided in Table 1. Females represented the largest part of the population (70 %). The ratio of female to male patients with a secondary education was 2:1, whilst the ratio of female to male patients with a tertiary education was 3:1. Regarding schooling, 95 % of patients had an education level of secondary school or higher.

**Table 1:** Demographics of the participants who participated in the survey.

Demographics			Patient Survey	Pharmacy survey
			*N* = 330 (% of *N*)	*N* *=* *16* (% of *N*)
Age group (years)	19–25		36 (11)	6 (38)
	26–55		221 (67)	10 (62)
	56–85		73 (22)	0
Gender	Male		95 (29)	7 (44)
	Female		233 (70)	8 (50)
	Other		2 (1)	1 (6)
Schooling level of patients	None	Males	1(0)	–
		Females	0	
		Other	0	
	Primary	Males	6 (2)	–
		Females	11 (3)	
		Other	0 (0)	
	Secondary	Males	48 (15)	–
		Females	95 (29)	
		Other	1 (0)	
	Tertiary	Males	40 (12)	–
		Females	127 (38)	
		Other	1 (0)	
Language spoken	Primary		63 (19)	–
	Secondary		267 (81)	–

- Question not relevant to the Pharmacists

The number of dispensing pharmacy staff that took part in the pharmacy survey was 17, however 1 response had to be discarded due to it being incomplete. In this study group, most participants were female and in the 26–55-year age group.

#### 4.1.2 Reading the PIL, PIL format preference and navigating the ePIL

Although the education level was deemed high, only 19 % of the total population read the PIL in its entirety. As to selective reading of sections, the top three sections read were ‘Possible side effects,’ ‘Dosage’ and ‘Indications for use.’ Patients reported a positive sentiment towards the ePIL with 80 % of the population preferring either the ePIL (35 %) or ePIL with a hardcopy (45 %). The ‘ePIL’ or ‘ePIL and hardcopy’ was also the preference for more than 70 % of patients who did not even read the paper PIL (Table 2*).* Of the dispensing pharmacy staff surveyed, 69 % preferred either the ePIL (31 %) or ePIL with a hardcopy (38 %) (Table 2).

**Table 2:** Patient information leaflet (PIL) format preference of patients and dispensing pharmacy staff who read the PIL as well as their ability to navigate the ePIL.

Survey	Patient Survey	Pharmacy Survey
Variable	*N* = 330 (% of *N*)	*N* *=* *16* (% of *N*)
Reading of the PIL		
Read the PIL (any amount)	243 (74)	–
Read the PIL in full	62 (19)	–
Only read the possible side effects	223 (68)	–
Only read section on dosage	203 (62)	–
Only read the indications for use	178 (54)	–
PIL format preference of participants		
ePIL	116 (35)	5 (31)
Hardcopy	67 (20)	5 (31)
ePIL+hardcopy	147 (45)	6 (38)
PIL format preference of participants who read the PIL (n_i_ = 243)		
ePIL	72 (30)	–
Hardcopy	47 (19)	–
ePIL+hardcopy	124 (51)	–
PIL format preference of participants who did not read the PIL (n_i_ = 87)		
ePIL	42 (48)	–
hardcopy	23 (27)	–
ePIL+hardcopy	22 (25)	–
Ability to navigate the ePIL upon scanning the QR code		
	n_i_ = 265	n_i_ = 9
Participants who found it easy to read the ePIL	257 (97) X^2^ = 114.16⁎⁎ *p* *<* 0.001 *C* = 0.487	8 (89) *p* = 0.0087 *C* = 0.746
Participants who found it easy to locate information on the ePIL	260 (98) X^2^ = 122.41⁎⁎ *p* *<* 0.001 *C* = 0.521	7 (78) *p* = 0.046 *C* = 0.630
Use of the ePIL to counsel patients		
		n_i_ = 9
Participants who would use the ePIL to counsel patients	–	7 (78) *p* = 0.3024 *C* = 0.337

- Question not relevant

X^2^ – Chi square.

*C –* Contingency coefficient.

⁎⁎ Correlation is significant at the 0.01 level (2-tailed).

More than 96 % of patients who scanned the QR code found it easy to read the ePIL (*C* = 0.487, *p* < 0.001) and locate the information needed (*C* = 0.521, *p* < 0.001) (Table 2). Almost 90 % of the dispensing pharmacy staff who scanned the QR code found it easy to read the ePIL (*C* *=* 0.746*, p* < 0.05*),* and 78 % confirmed they could locate the required information on the ePIL (*C* *=* *0.630, p* < 0.05*)* and would utilise the ePIL to counsel patients (Table 2).

#### 4.1.3 Access to technology

When assessing patients' access to technology, knowledge of QR codes, and ability to scan a QR code, a smaller proportion (41 %) in the older age group of 56–85-years were knowledgeable on QR codes compared to the younger age groups of 19–25 (72 %) and 26–55-years (68 %). There was a moderate measure of association between age and access to the internet, devices and data i.e. *C* = 0.285, *C* = 0.282, *C* = 0.292, respectively (Table 3). The younger age group was more willing and able to scan the QR code and required less assistance to scan the QR code. Overall, 85 % of patients were willing and 80 % able to scan the QR code. Both variables displayed a strong contingency coefficient and a high significance (*p* < 0.001) (Table 3).

**Table 3:** Indication of participants who had access to technology, as well as their knowledge and ability to scan the QR code.

Patient Survey (N = 330)							Pharmacy Survey (N = 16)			
Variable	n_i_	Age group	% of n_i_	X^2^-value	*C*-value	% of *N*	n_i_	Age group	% of n_i_	*% of N*
Access to the internet	n_i_ = 36	19–25	97	29.16⁎⁎	0.285	87	n_i_ = 6	19–25	33	69
	n_i_ = 221	26–55	91				n_i_ = 10	26–55	90	
	n_i_ = 73	56–85	69							
Access to a device	n_i_ = 36	19–25	100	28.89⁎⁎	0.282	91	n_i_ = 6	19–25	83	44
	n_i_ = 221	26–55	95				n_i_ = 10	26–55	20	
	n_i_ = 73	56–85	75							
Access to data	n_i_ = 36	19–25	94	30.76⁎⁎	0.292	89	–	–	–	–
	n_i_ = 221	26–55	94				–	–	–	
	n_i_ = 73	56–85	71							
Knowledge of QR codes	n_i_ = 36	19–25	72	18.43⁎⁎	0.230	63	n_i_ = 6	19–25	83	63
	n_i_ = 221	26–55	68				n_i_ = 10	26–55	50	
	n_i_ = 73	56–85	41							
Willingness to scan the QR Code	n_i_ = 36	19–25	94	36.63⁎⁎	0.316	85	n_i_ = 6	19–25	50	56
	n_i_ = 221	26–55	91				n_i_ = 10	26–55	60	
	n_i_ = 73	56–85	63							
Able to scan the QR Code	n_i_ = 36	19–25	97	37.21⁎⁎	0.318	80	n_i_ = 6	19–25	67	56
	n_i_ = 221	26–55	86				n_i_ = 10	26–55	50	
	n_i_ = 73	56–85	56							
Assistance needed to scan the QR code	n_i_ = 36	19–25	28	34.02⁎⁎	0.306	46	n_i_ = 6	19–25	17	13
	n_i_ = 221	26–55	39				n_i_ = 10	26–55	10	
	n_i_ = 73	56–85	75							

- Question not relevant.

X^2^ – Chi square.

*C –* Contingency coefficient.

There was no association between access to technology and age groups in the pharmacy survey and therefore data is not included.

There was no association between QR codes knowledge, scanning the QR codes and age groups in the pharmacy survey and therefore data is not included.

⁎⁎ Correlation is significant at the 0.01 level (2-tailed).

Sixty-three percent of the dispensing pharmacy staff who participated in the survey were aware of what a QR code was and 69 % had access to the internet at the pharmacy (Table 3). More than 55 % of this group were willing and able to scan the QR code with 13 % needing assistance.

The association between ease of scanning the QR code and age groups indicated that the younger age groups found it “very easy” to scan the QR code versus the group aged 56–85 years (X^2^ = 49.32, p < 0.001, *C* = 0.361) (Table 4). Of the latter, approximately a third indicated that it was “hard” to scan the QR code, with 75 % requiring assistance to do so (Table 3, Table 4). More than 75 % of the patient population found it “very easy” or “easy” to scan the QR code (Table 4). Patients with no education or only primary school level education needed more assistance (100 % and 82 %, respectively) to scan the QR code compared to those who had secondary (55 %) or tertiary (34 %) education (Table 4). Furthermore, only 26 % of patients who previously scanned a QR code needed assistance to scan the QR code, and there was a strong association between “assistance required to scan the QR code” and “experience of previously scanning a QR code” (Table 4; *p* < 0.001, *C* = 0.424).

**Table 4:** Ability to scan the quick response code and the correlation with age and schooling level.

Variable		Total (n_i_)	Ease of scanning the QR code⁎ % of n_i_					X^2^-value	*C*-value
			1	2	3	4	5		
Age group	19–25	36	69	25	3	3	0	49.32⁎⁎	0.361
	26-55	221	55	28	2	10	5		
	56–85	73	26	27	6	38	3		
Total population		330	50	27	3	15	5		
Variable		Total (n_i_)	Assistance required to scan the QR code (% of n_i_)					X^2^-value	*C*-value
Schooling level	None	1	1 (100)					24.61⁎⁎	0.263
	Primary	17	14 (82)						
	Secondary	144	79 (55)						
	Tertiary	168	56 (34)						
Previous experience of scanning a QR code	Yes	191	50 (26)					72.15⁎⁎	0.424

X^2^ – Chi square.

*C –* Contingency coefficient.

⁎ Ease of scanning the QR code: 1 = Very easy, 2 = Easy, 3 = Okay, 4 = hard, 5 = very hard.

⁎⁎ Correlation is significant at the 0.01 level (2-tailed).

#### 4.1.4 Willingness and ability to scan the QR code

The percentage of male versus female patients willing to scan the QR code was similar (Table 5). However, for the dispensing pharmacy staff, most males (86 %) were willing and able (71 %) to scan the QR code compared to their female counterparts. None of the male pharmacy staff required assistance to scan the QR code (Table 5).

**Table 5:** An indication of the willingness, ability and assistance to scan the quick response code.

	Patient Survey				Pharmacist Survey			
QR code	Male n_i_ = 95 (% of n_i_)	Female n_i_ = 233 (% of n_i_)	Other n_i_ = 2 (% of n_i_)	*N* = 330 (% of N)	Male n_i_ = 7 (% of n_i_)	Female n_i_ = 8 (% of n_i_)	Other n_i_ = 1 (% of n_i_)	*N* = 16 (% of N)
Willing to scan	77 (81)	202 (87)	2 (100)	281 (85)	6 (86)	2 (25)	1 (100)	9 (56)
Able to scan	69 (73)	194 (83)	2 (100)	265 (80)	5 (71)	4 (50)	0	9 (56)
Assistance needed to scan	40 (42)	112 (48)	0	152 (46)	0	1 (13)	1 (100)	2 (13)

Cronbach's alpha for all scale level questions in the patient survey was >0.76.

Cronbach's alpha for all scale level questions in the pharmacy survey was >0.75.

### 4.2 Regulatory affairs pharmacists focus group

Four aspects were evident in the regulatory affairs pharmacist group (Table 6). Their familiarity with the QR code; all participants were familiar with QR codes and able to cite examples. Current usage of the QR code; some pharmacists were able to quote examples of using the QR code in current practice. All the regulatory affairs pharmacists preferred the ePIL only. Whilst some pharmacists were concerned about older patients not being familiar with how to use their smartphone to scan a QR code, others indicated, that patients could be taught on how to scan the QR code. There was a concern whether patients in rural areas would have a smartphone.

**Table 6:** Themes and quotes from the regulatory affairs pharmacists focus group study.

Theme	Quotes
Theme 1: Familiarity with the QR code	“*QR codes are present for banking apps.*” “*During COVID most restaurant implemented QR codes for their menus.*” “Y*ou see lots of adverts with QR codes.*” “A*pplication forms can now be access using a QR code.*” “E*verything is going electronic.*” *“Used in supply chain for track and trace implementation.”*
Theme 2: Current usage of QR codes in the pharmaceutical industry	“*We are using it already for providing medical information and managing Dear HCP letters.”* “*It is a requirement for products on tender to include a 2D bar code in the supply chain to aid tracking of the product.”* “We *are utilizing QR codes to capture the PI/PIL on marketing material*.” *“Used in batch traceability to prevent counterfeiting.”* *“OTC industry in South Africa is using it for PI and PILs”*
Theme 3: Regulatory Affairs Pharmacists' preference	*“I would prefer the QR code* versus *100 pieces of paper,”* *“It will require a mind shift change on the part of the patient”* *“There will always be those patients who prefer paper.”* “*I would be delighted to only have an ePIL.”* *“Pharmacy personal could offer the services to print the PIL for the patient wanting the paper PIL*.” Quotes from focus group: theme 3: Advantages of using QR codes for PILs *“It is easy to use.”* *“Enables quick access to the PIL when needed as most people lose the PIL.”* *“Makes it easier to read the PIL as older people can increase the size of the text.”* “*It is a more efficient way of managing ‘Dear Doctor letters’ as well as provision of updated product and safety information as well as medical information.”* “*It supports sustainability as it reduces the need to place a printed leaflet in the product pack.”* *“It reduces paper wastage.*” “*Allows for shared packs across many countries as the QR code can capture more than one language.*” “*There are many products dispensed without a PIL particularly in the State sector, the QR coded PIL will be extremely beneficial here.”* *“May help the hospital setting where medication is dispensed without the leaflet or patients not counselled on how to use the product.”* *“QR codes can be used by the pharmacy to provide an ePIL when breaking a pack to dispense to a patient as these packs do not come with a leaflet.”*
Theme 4: Debate over technology and the ability to scan the QR code	“*What about older patients who may not have a smartphone to scan the QR code?*” “*The dispensing pharmacist or family can teach them how to use their smartphone to scan the QR code. I showed my mum how to use her smartphone.*” “W*e need to give patients credit and not to assume patients are ignorant or do not have a smartphone.”* *“We need to consider patients in rural areas who may not have smartphones.”*

## 5 Discussion

This study is the first in South Africa to assess the feasibility of ePILs among patients, dispensing pharmacy staff as well as regulatory affairs pharmacists. The objectives of the patient study were met by including a wide age range (18–85 years) inclusive of both men and women with the majority having taken English to at least secondary/tertiary education level (95 %). (Table 1).

The results of the patient study indicated that age had a highly significant relation (*p* < 0.001) and a moderate strength of association with knowledge of the QR code (*C* = 0.230) (Table 3). The older age group was less familiar with QR codes compared to the younger age groups in both the patient and pharmacy surveys. This corroborated with studies carried out in the United States, Ireland and India.14., 15, 16 The older patient age group (56–85-years) had reduced access to the internet, a mobile device and mobile data, while just over a third of this group found it ‘hard’ to scan the QR code with the majority of these participants needing assistance. (Table 3). This point of concern was also raised by the regulatory affairs pharmacists. This analysis supports the digital divide between younger and older patients. Younger patients are more familiar, knowledgeable and early adopters of technology such as smartphone use for banking apps, online purchase of items, accessing the internet etc. versus the older patients who may not be familiar with the technology and hesitant to adopt it due to a lack of trust and privacy issues.^17^, ^18^, ^19^, ^20^, 21., 22

In addition to some older patients who needed assistance to scan the QR code, patients with no or only primary schooling also required assistance (Table 3, Table 4). There was a significant (*p* < 0.001) and strong association (*C* = 0.424) between assistance required to scan the QR code and experience of previously scanning a QR code (Table 4), indicative that patients can be educated on how to scan the QR code as proposed by Choudrie et al.^20^ and Wang.^21^

Overall, 46 % of the study population needed assistance to scan the QR code (Table 3), which is more than double the number of patients needing assistance in the study carried out at Queen Victoria Hospital in the United Kingdom (UK), indicating a possible lag in the adoption of the technology in Tshwane.^23^

The roll out of ePILs will require patient education focusing on older and uneducated patients, on how to scan QR codes.^6,14-15,17–18.^ This was also a suggestion from the focus group. The PIL may need to be printed to support the transition. The South African Pharmacy Council, together with industry associations such as the South African Association of Community Pharmacists have a key role to play in ensuring pharmacy staff support patients' transition to ePILs in this regard.^24^ The current results indicated that pharmacy staff who did scan the QR code would in fact use the ePIL to counsel patients, a key requirement of the Good Pharmacy Practice Guidelines.^25^

Irrespective of age and gender, both the patient and pharmacy survey demonstrated that 63 % (Table 3) of participants were knowledgeable about QR codes. Some regulatory affairs pharmacists in the focus group study indicated that they were already using the QR codes in practice such as the provision of medical information, managing ‘Dear Healthcare Professional letters,’ in the supply chain to track the product and prevent counterfeiting, and for easy access to PIs and PILs on marketing material. Most patients (85 %) were willing, and 80 % were able to scan the QR code to access the ePIL (Table 5) with similar results shown for the male pharmacy staff (Table 5; 86 % were willing and 71 % able to scan the QR code), however overall, the dispensing pharmacy staff where a bit apprehensive to scan the QR code with only 56 % willing and able to scan the QR code. This could be related to privacy concerns. A more detailed study would be required to determine the exact reasons.

Compared to the study carried out at Queen Victoria Hospital in the UK, 81 % of patients found it very easy/easy/okay to scan the QR code at Tshwane District versus 96 % of patients in the UK, again indicating a possible lag in the adoption of the technology in Tshwane, leading to participant unfamiliarity.^23^

Irrespective of age and gender, the results of both the patient and pharmacy survey revealed a high percentage preference for either the ‘ePIL’ or ‘ePIL with hardcopy,’ i.e. 80 % and 69 % respectively (Table 2). Patients who did not even read the current paper PIL also preferred either the ‘ePIL’ or ‘ePIL with hardcopy,’ i.e. 73 %. The data in this study is therefore a strong indicator of a positive sentiment towards ePILs, be it alone or together with a hardcopy (Table 2) as a complementary tool as suggested by the Pharmaceutical Group of the 10.13039/501100000780European Union (EU).^4^^,^^5^^,^^26^ Patients could be transitioned to ePILs by providing both the ePIL and the hardcopy prior to full implementation of ePILs.^5^^,^^6^ This dual system is supported by countries in the EU, Canada, Brazil and the United States.^5^ Whilst countries of the Gulf Cooperation Council such as Bahrain, Kuwait, Oman and Qatar support the ePIL by means of QR codes before the end of 2025.27., 28., 29., 30.

Based on the patient's education levels, it was expected that they would have an adequate literacy level (i.e. ability to read and write) to read the PIL. Even though 95 % of the patient population had secondary or higher education, which correlated with the South African General Household survey of 2021,^31^ only 19 % read the PIL in full.There are various reasons why patients would not want to read the paper PIL; long length of the PIL which is time consuming to read; small font size and closely spaced sentences, which makes reading difficult; only being interested in certain sections of the PIL or difficulty in understanding the text or language.^32^^,^^33^ By design, the ePIL conveniently allows the patient to locate the section of interest (scrolling down text or using search function).^8^^,^^34^ In this study more than 95 % of the patients found it easy to read the ePIL and locate the information they sought. This finding was echoed by the pharmacists and supported by the focus group.

For 81 % of the population, English was a secondary language. The current legal requirement in South Africa is to have the PIL available in English and one other language. South Africa has 11 official languages, and translation of the PIL into any other language than English may not result in the correct medical translation given that not all the medical taxonomy is available in the different official languages.^35^^,^^36^

A key requirement for ePILs includes access to a smartphone or mobile scanning application to scan the QR code to access the ePIL.^9^ The results indicated over 87 % of patients had access to the internet, a mobile device, and mobile data. This finding closely correlates with data from the Independent Communications Authority of South Africa (ICASA), reporting that 85 % of users in Gauteng used a smartphone to access the internet and is also backed by the General Household survey of 2021, indicating that over 90 % of households in Gauteng exclusively use cellular phones to access the internet and therefore indicative they have data. The ICASA report of March 2024 indicated ∼70 % of households nationally had access to the internet through a mobile device with metropolitan areas sitting at 74 % and rural areas at 61 %.^37^^,^^38^ To further promote access to the internet, ICASA in 2022, made zero-rating of government and public benefit organizations digital content a condition of license thereby promoting free access to websites to students in support of their studies and to the public ensuring access to vital health information. In addition, on 5 April 2024, the South African government announced acceleration of the program to connect citizens to the internet offering low-cost data at an affordable rate of ZAR5 a day per Gig of data.39., 40, 41.

In the midst of the fourth industrial revolution, the transition to a digital society is rapidly accelerating. In the pharmaceutical industry, this includes the move to provide ePILs as replacement of the hardcopy PIL.^42^^,^^43^ Advantages of the ePIL over the hardcopy PIL for the consumer include the ability to read the ePIL through increasing the size of the text accommodating patients with visual disturbances, and access to the PIL at any place or when travelling due to the portability of a smartphone/device.^24^^,^^26^^,^^34^^,^^44^ Inclusion of audio excerpts is also possible through the QR code making ePIL more inclusive for all patients.^24^ From the manufacturer and regulators side the key advantage of the ePIL is the ability to update the ePIL in real time with new information. Should this be applicable to dosing or interactions of the product, this could impact significantly to decrease morbidity and even mortality. Another important impact in eliminating the hardcopy PIL is; the reduction of the carbon foot print, paper wastage (trash), chopping down of trees, contributing to a cleaner environment as there will be no need to discard old leaflets, aligning to the United Nations and global Sustainable Development Goal (SDG) 12; responsible production and consumption, SDG 13; climate action and goal, SDG 15; life on land, ensuring a cleaner, healthier and sustainable environment.^5^^,^^6^^,^^8^^,^^23^^,^^24^^,^^26^^,^44., 45., 46.

In addition to the advantages of an ePIL mentioned by the United States Food and Drug Agency (US FDA), European Medicines Agency (EMA) and Self-care Association of South Africa (SCA), regulatory affairs pharmacists articulated the added advantage of using QR codes to deliver ePILs for patient-ready packs which are dispensed without a leaflet either in the public hospital or private pharmacy. Clearly the benefits of an ePIL through a QR code surpass the hardcopy PIL.

Patients, dispensing pharmacy staff, and regulatory affairs pharmacists prefer a format with ePIL availability through a QR code, indicating a possible transition and readiness for all parties.

The South African Health Products Regulatory Authority (SAHPRA), with the support of the SCA, has commenced the building of an online directory for Over-the Counter (OTC) SAHPRA-approved medicines. The website provides key information on OTC medicines, including the PI and PIL and therefore, the website which is expected to be expanded for prescription medicines and could be utilized to generate a QR code for the PI/PIL.^47^

### 5.1 Limitations of the study

The study was limited to a district hospital. A study in a wider population covering several hospitals, both at the academic and district level in both urban and rural areas is necessary to draw conclusively on both pharmacy staff as well as patient knowledge of QR codes, use of smartphones to scan the QR code and PIL format preference.

## 6 Conclusion

Electronic patient information leaflets have the potential to revolutionise the healthcare industry. This study showed that patients, dispensing pharmacy staff, and regulatory affairs pharmacists are ready to start the transition. Whilst regulation 12 of the Medicines Act, related to the PIL has been amended by SAHPRA supporting ePILs for a second language, industry has the opportunity to comment on these regulations to facilitate transition to ePILs.

SAHPRA and the South African Pharmacy Council, together with industry associations such as the South African Association of Community Pharmacists, have a key role to play in terms of providing patients with training on how to scan the QR-coded PIL and locate the information needed. This will aid in patient adoption of the innovative technology, facilitating the full role out of paperless PILs.

## Funding

This research did not receive any specific grant from funding agencies in the public, commercial, or not-for-profit sectors.

## Acknowledgment of individuals who were of direct help in the preparation of the study

The patients and staff at the Tshwane District Hospital.

The pharmacy fraternity.

## CRediT authorship contribution statement

**Githa Singh:** Writing – review & editing, Writing – original draft, Resources, Project administration, Methodology, Investigation, Formal analysis, Data curation, Conceptualization. **Sarel J. Brand:** Writing – review & editing, Supervision. **Vanessa Steenkamp:** Writing – review & editing, Supervision.

## Declaration of competing interest

None.
